# PAR4 misexpression in the cerebrovasculature drives excess fibrin deposition in 5XFAD mouse and cognitive decline in AD patients

**DOI:** 10.1002/alz70861_108183

**Published:** 2025-12-23

**Authors:** Kevin E. Erreger, David E. Reyes, Jared Phillips, Yanling Wang, Julie A Schneider, David A. A. Bennett, Matthew Schrag, Timothy J. Hohman, Heidi E. Hamm

**Affiliations:** ^1^ Vanderbilt University, Nashville, TN USA; ^2^ Department of Pharmacology, Vanderbilt University, Nashville, TN USA; ^3^ Rush University, Chicago, IL USA; ^4^ Rush Alzheimer's Disease Center, Rush University Medical Center, Chicago, IL USA; ^5^ Rush Alzheimer’s Disease Center, Department of Neurological Sciences, Rush University Medical Center, Chicago, IL USA; ^6^ Vanderbilt University Medical Center, Nashville, TN USA; ^7^ Vanderbilt Memory & Alzheimer’s Center, Vanderbilt University Medical Center, Nashville, TN USA

## Abstract

**Background:**

Platelet activation may be a central mediator of a chain of events leading to microinfarcts, leakage of thrombin and fibrin through the blood‐brain barrier and chronic neuroinflammation that typify Alzheimer’s Disease (AD). The platelet thrombin receptor Protease Activated Receptor 4 (PAR4) is responsible for platelet activation and amplification of thrombin generation. Thrombin cleaves fibrinogen to fibrin, and pathologic fibrin deposition in the cerebral microvasculature is itself a risk factor for Alzheimer’s disease. Fibrin interaction with vascular amyloid β (Aβ) leads to degradation‐resistant blood clots. This process initiates inflammation both in the vessel and adjacent parenchyma. Inflammation in general has been reported to turn on expression of PAR4 in endothelial cells not typically expressing PAR4.

**Method:**

1) Data were acquired from the Religious Orders Study (ROS) and the Rush Memory and Aging Project (MAP) to study the correlation of the PAR4 gene expression or methylation with AD diagnosis and longitudinal cognitive decline. 2) 5xFAD amyloid model mice were crossed with PAR4KO mice to test for pathology and markers of inflammation.

**Result:**

1) PAR4 gene *F2RL3* mRNA was elevated in AD cases and was associated with worse retrospective longitudinal cognitive performance. We also report a significant association of *F2RL3* epigenetic demethylation with cognitive decline. 2) 5xFAD mice exhibit increased PAR4 protein expression on vascular endothelial cells. 5xFAD mice exhibit increased vascular fibrin deposits compared to WT mice, and this fibrin deposition is reduced in 5xFAD/PAR4KO compared to 5xFAD.

**Conclusion:**

In a human study the PAR4 gene *F2RL3* mRNA expression is associated with multiple AD‐relevant outcomes and its encoded product, PAR4, may play a role in disease pathogenesis. PAR4 is a platelet receptor that functions upstream of fibrin deposition in 5xFAD amyloid mice, leading to misexpression in the microvasculature. Fibrin deposits have been reported to be inflammatory in 5xFAD and future work will focus on teasing apart the contribution of PAR4 mediated fibrin deposits in inflammation and microglial activation.

